# Identification of a novel TSC2 c.3610G > A, p.G1204R mutation contribute to aberrant splicing in a patient with classical tuberous sclerosis complex: a case report

**DOI:** 10.1186/s12881-018-0686-6

**Published:** 2018-09-20

**Authors:** Ruixiao Zhang, Jianhong Wang, Qing Wang, Yue Han, Xuejun Liu, Irene Bottillo, Yanhua Lang, Leping Shao

**Affiliations:** 1 Department of Nephrology, the Affiliated Qingdao Municipal Hospital of Qingdao University, No.5 Donghai Middle Road, Qingdao, 266071 People’s Republic of China; 2 Central Laboratory, the Affiliated Hospital of Qingdao University, Qingdao, 266003 People’s Republic of China; 3 Organ Transplantation Center, the Affiliated Hospital of Qingdao University, Qingdao, 266003 People’s Republic of China; 4 Department of Ophthalmology, the Affiliated Hospital of Qingdao University, Qingdao, 266003 People’s Republic of China; 5 Department of Radiology, the Affiliated Hospital of Qingdao University, Qingdao, 266003 People’s Republic of China; 6 Division of Medical Genetics, Department of Molecular Medicine, Sapienza University, San Camillo-Forlanini Hospital, 00185 Rome, Italy

**Keywords:** Tuberous sclerosis complex, *TSC1*, *TSC2*, Novel mutation, Aberrant splicing

## Abstract

**Background:**

Tuberous sclerosis complex (TSC) is an autosomal dominant disorder characterized by hamartomas in any organ systems. Mutations in the *TSC1* or *TSC2* gene lead to the dysfunction of hamartin or tuberin proteins, which cause tuberous sclerosis complex.

**Case presentation:**

We describe the clinical characteristics of patients from a Chinese family with tuberous sclerosis complex and analyze the functional consequences of their causal genetic mutations. A novel heterozygous mutation (c.3610G > A) at the last nucleotide of exon 29 in *TSC2* was identified. On the protein level, this variant was presumed to be a missense mutation (p.Gly1204Arg). However, the splicing assay revealed that this mutation also leads to the whole *TSC2* exon 29 skipping, besides the wild-type transcript. The mutated transcript results in an in-frame deletion of 71 amino acids (p.Gly1133_Thr1203del) and its ratio with the normal splice product is of about 44:56.

**Conclusions:**

The novel c.3610G > A *TSC2* mutation was identified in association with tuberous sclerosis complex. And it was proven to code both for a missense-carrying transcript (56%), and for an isoform lacking exon 29 (44%).

**Electronic supplementary material:**

The online version of this article (10.1186/s12881-018-0686-6) contains supplementary material, which is available to authorized users.

## Background

Tuberous Sclerosis Complex (TSC) is a neurocutaneous disorder with a birth incidence of approximately 1/6,000–1/10,000, and affecting almost 2 million people worldwide [1, 2]. It is characterized by the uncontrolled benign mesenchymal tumor growth in multiple organ systems, including the brain, skin, heart, lung and kidney [3, 4]. The spectrum of clinical manifestation is very wide among individuals due to the different location of the lesions, with combination of symptoms including seizures, developmental delay, intellectual disability, behavioral problems, skin abnormalities, and lung or kidney disease. The differences between gender and races of TSC have not been found, and it will appear at any age ranging from birth to adulthood [5]. TSC is an autosomal-dominantly inherited disorder with high penetrance but variable expressivity, regardless of mutations in either *TSC1* or *TSC2* gene.

*TSC1* and *TSC2* respectively code for the hamartin and tuberin proteins, which act as tumor growth suppressors. Hamartin and tuberin interact with each other through their coiled-coil domains to form a stable and functional heterodimer that promotes the GTPase activity of Rheb protein, thus preventing the Rheb-GTP-dependent stimulation of cell proliferation, adhesion, growth, differentiation and migration, through the mTOR pathway [3, 6–8]. Consistent with the Knudson ‘two-hit’ tumor-suppressor gene model [9], a first germline mutation inactivating one allele of either *TSC1* or *TSC2*, and subsequently a somatic event (often loss of heterozygosity) inactivating the second allele, result in upregulation of the mTOR pathway and lead to overactive cell growth and proliferation [10].

So far, 1,478 different mutations in the *TSC* genes have been identified to be responsible for the vast majority of cases of TSC according to HGMD (HGMD Professional 2017.4). Herein, we performed clinical and genetic investigation in a Chinese family with classical TSC, and reported the novel heterozygous c.3610G > A, p.G1204R mutation in exon 29 of *TSC2* gene, Meanwhile, the effect of this variant in the splicing process was investigated by a mini-gene assay and the RNA analysis.

## Case presentation

**Fig. 1:**
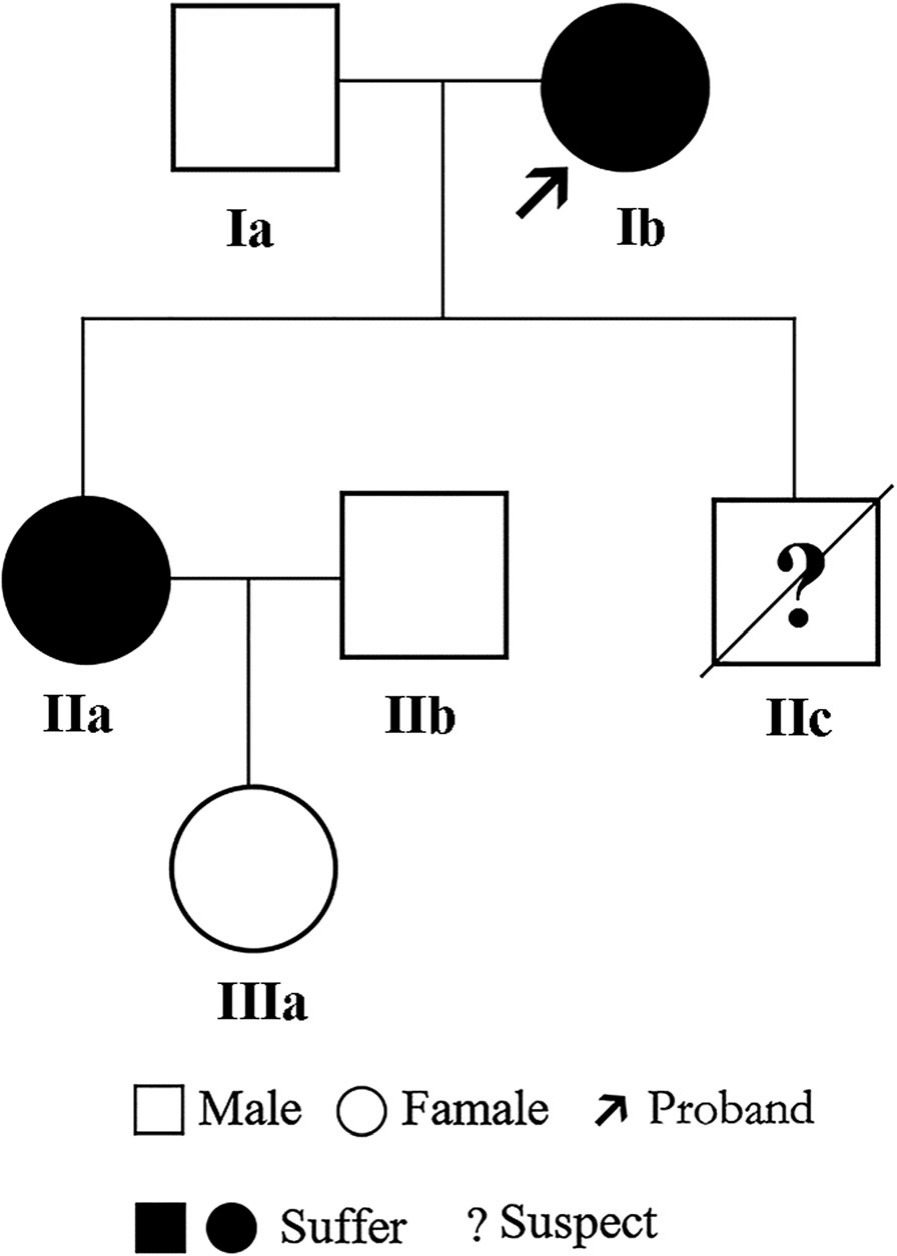
Pedigree of the kindred with Tuberous Sclerosis

**Fig. 2:**
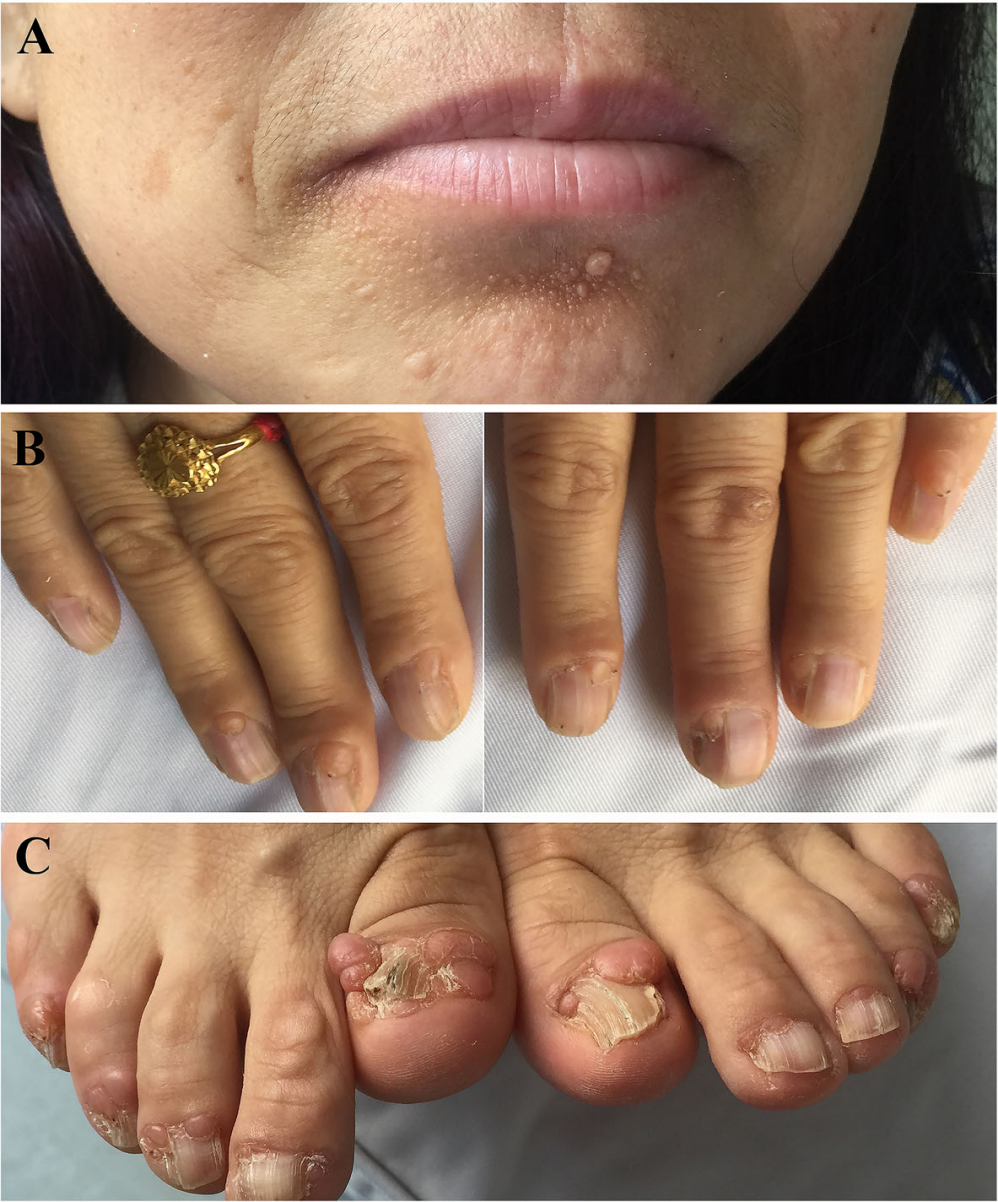
Clinical photographs of the proband. **a**. adenoma sebaceum; **b**, **c**. subungual fibromas

**Fig. 3:**
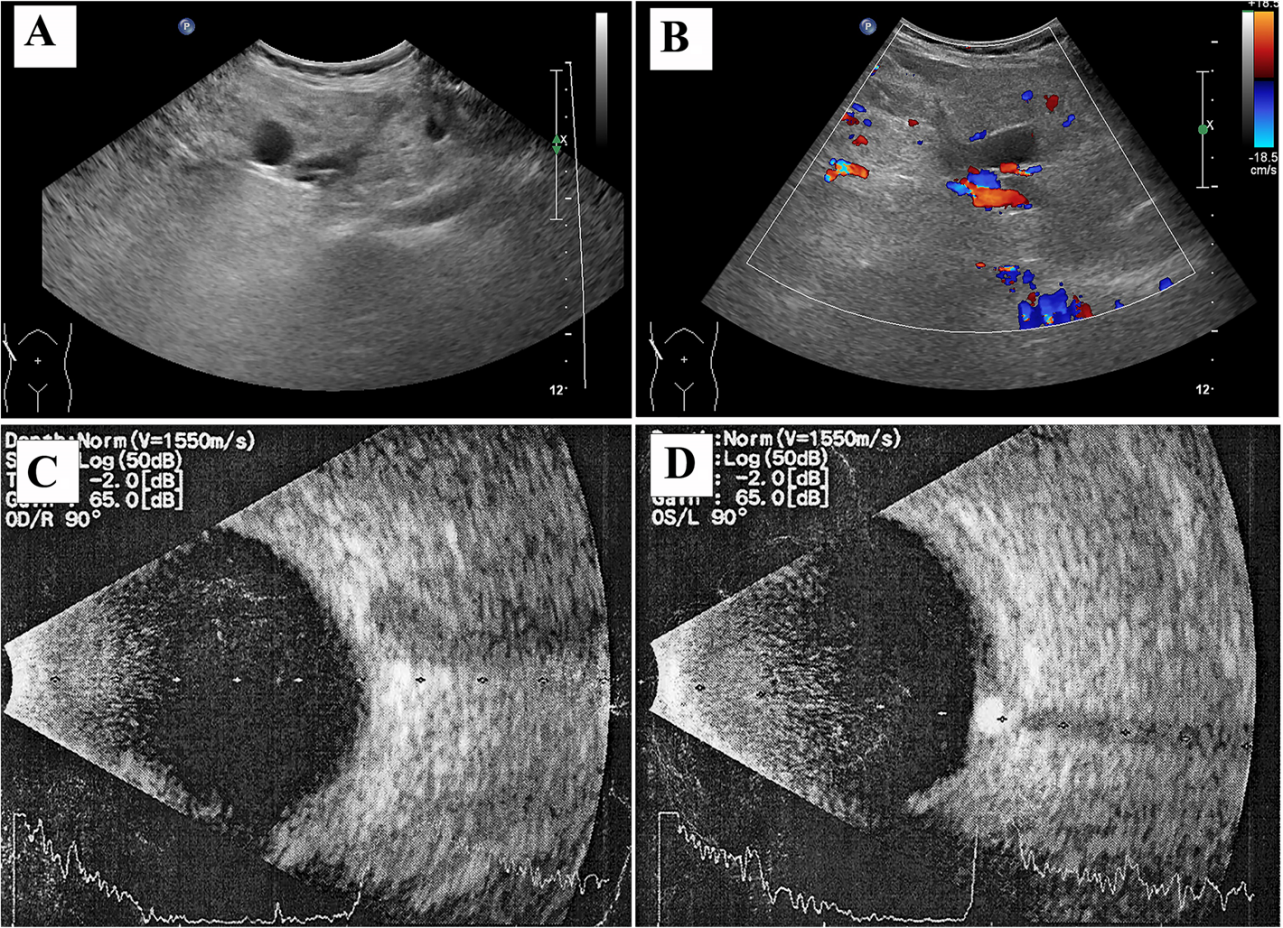
Ultrasonography of both kidneys and eyes of the proband. **a**, Renal ultrasound image showing a heterogenous mass with a large echogenic fatty component and a less echogenic soft-tissue component with prominent vessels within it; **b**, Color doppler ultrasoud image showing highly vascular fatty masses; **c**, Right eye; **d**, Left eye: demonstrating a hyperechogeneic mass (hamartoma) with posterior shadowing due to calcifications

**Fig. 4:**
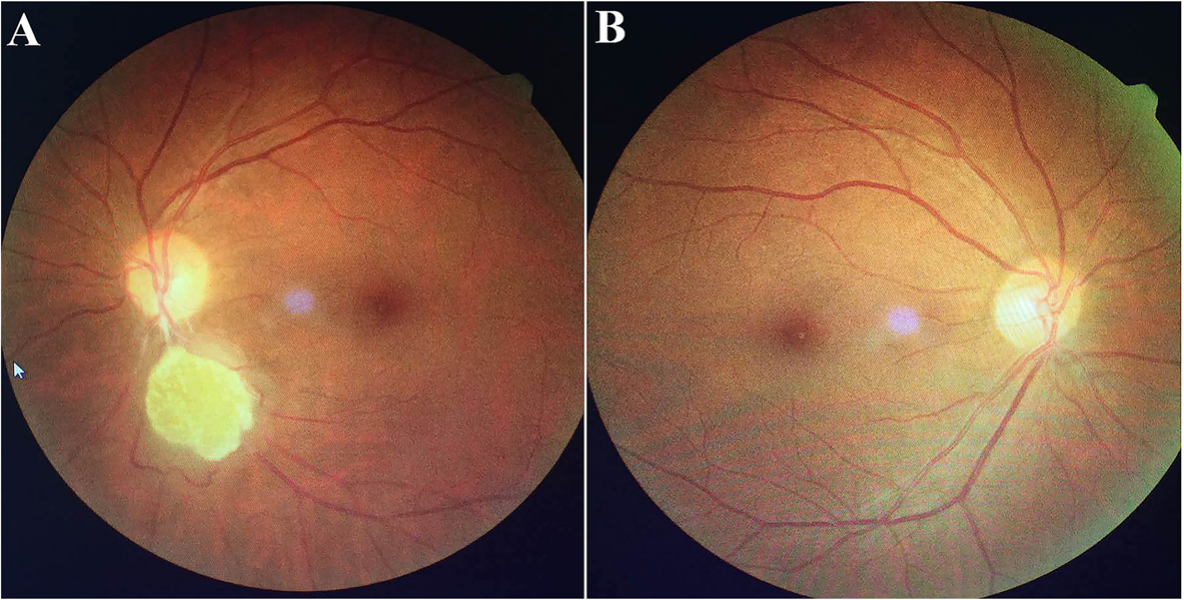
Fundus Photograph of the proband Ib. **a**. Left eye, Fundus photograph of a hamartoma with central calcifications and a surrounding translucent zone. **b**. Right eye

**Fig. 5:**
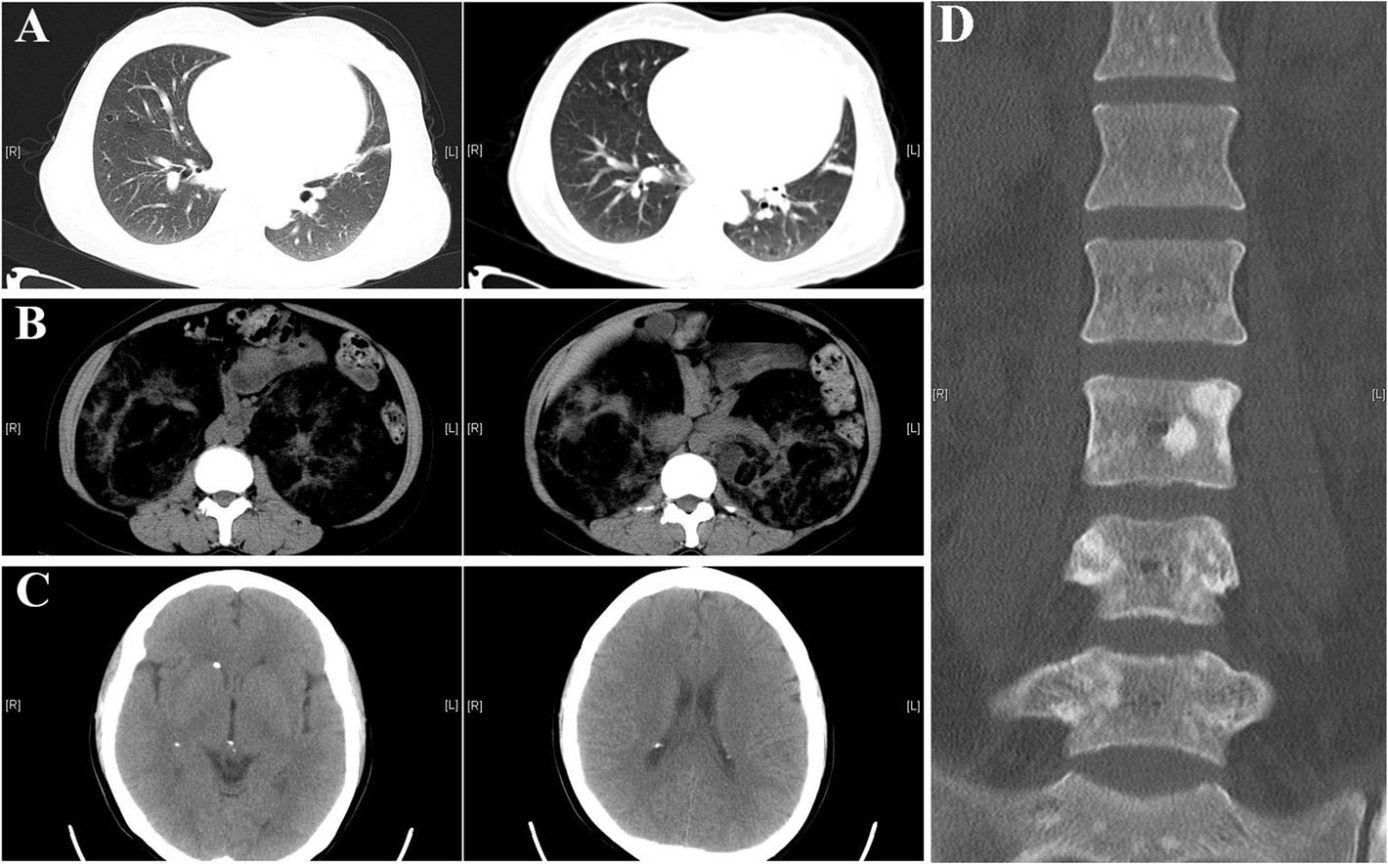
Non-contrast-enhanced CT Imaging of the proband Ib. **a**. Lung CT scans showing presence of multiple variable sized air filled cysts consistent with lymphangiomyomatosis. **b**. Abdomen CT scans showing massive bilateral predominantly fatty renal masses consistent with angiomyolipomas. **c**. Brain CT showing multiple calcified subependymal nodules. **d**. Spine X-ray showing multiple patchy sclerotic lesions

The pedigree of the family is shown in Fig. 1. The proband (Ib in Fig. 1) was a 47-year-old female of Han Chinese ethnicity (with family from Shandong province, China), who was admitted to our hospital with a chief complaint of “fatigue and chest tightness for 7 days”. She never had a similar episode before. She had neither intellectual disability nor a history of seizures. Her blood pressure was 135/85 mmHg. Physical examination revealed typical facial angiofibromas (adenoma sebaceum) and multiple periungual fibromas (Fig. 2), and obvious abdominal distention. Her biochemical laboratory test demonstrated renal insufficiency (serum creatinine levels: 3.1 mg/dL, normal values 0.5–1.1 mg/dL; estimated glomerular filtration rate: 17.1 ml/min/1.73 m^2^, estimated by CKD-EPI formula) and moderate anemia (hemoglobin of 8.6 g/dL, normal values 11–15 g/dL). Ultrasonography of her both kidneys manifested a heterogeneous mass with a large echogenic fatty component and a less echogenic soft-tissue component with prominent vessels within it, which was suggestive of giant bilateral renal angiomyolipomas (AML) (Fig. 3a). Highly vascular fatty masses were seen on Color Doppler scans (Fig. 3b). Ultrasonography of the left eyes demonstrated a hyperechogenic lesion with posterior shadowing due to calcifications (Fig. 3d). Fundus photograph showed a hamartoma with central calcifications and a surrounding translucent zone in the left eye, suggesting the retinal hamartoma (Fig. 4). Thereafter, non-contrast-enhanced computed tomography (CT) of the abdomen confirmed the ultrasonography diagnosis of giant bilateral renal AMLs with prominent fatty components and internal prominent vessels. Multiple variable-sized air-filled cysts throughout the parenchyma were noted in both lungs consistent with lymphangioleiomyomatosis (LAM) on lung CT. And brain CT revealed multiple calcified subependymal nodules. Images of spine displayed multiple patchy sclerotic lesions (Fig. 5).

Therefore, we considered that the patient fulfilled the diagnostic criteria of TSC [11]. The patient accepted the treatment with the mTOR inhibitor everolimus (10 mg/d) because her AMLs nearly invaded all tissues of bilateral kidneys which could not be removed completely. Two months later, the patient felt a significant alleviation in the feeling of abdominal swelling. Regrettably, she had to discontinue her treatment because of financial difficulties. After half a year follow-up, the patient remained in a stable condition. Besides, she had a son (IIc) who died of epilepsy at the age of six. Her daughter (IIa) also had the presentation of typical facial angiofibromas and multiple periungual fibromas, but she denied further imaging examination. Her husband (Ia) and granddaughter (IIIa) did not show any significant symptoms or abnormalities on testing. To further confirm the diagnosis of TSC, mutation analysis of the *TSC1* and *TSC2* gene and bioinformatics analysis were performed by the methods in Additional file 1. The CARE guidelines were followed in reporting this case.

**Fig. 6:**
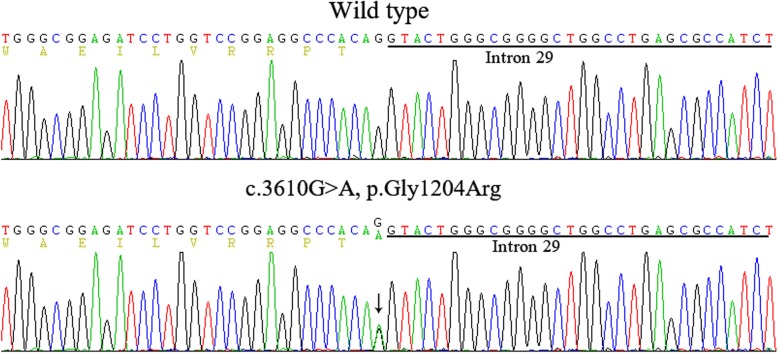
TSC2 mutations identified in the proband Ib with TSC

By the next generation sequencing, the proband was found to carry a heterozygous guanine to adenine substitution of the last nucleotide in exon 29 of *TSC2* (c.3610G > A), resulting in a single amino acid substitution from Glycine to Arginine at amino acid position 1204 (p.Gly1204Arg) (Fig. 6). Sanger sequencing validation of all family members revealed that the proband’s daughter (IIa) carried the same heterozygous p.Gly1204Arg mutation in *TSC2* gene, whereas other family members and 100 unrelated controls did not harbor this mutation. No mutation was identified in *TSC1* gene.

The c.3610G > A variant was considered as novel since it was neither present in ExAC, 1000G, nor in HGMD databases (HGMD Professional 2017.4). However, another substitution at the same codon (c.3611G > A, p.Gly1204Glu) has been reported to be related with TSC [12].

The p.Gly1204Arg alteration was predicted as deleterious by three different softwares (i.e. MutationTaster, SIFT and PolyPhen-2). Moreover, the p.Gly1204Arg variant is highly conserved among 8 different species (human, *Callorhinchus milii*, *Macaca mulatta*, chimpanzee, house mouse, rat, xenopus tropicalis and zebrafish) by using Vector NTI Advance 10-Align.

It was noteworthy that the c.3610G > A was a nucleotide substitution at the last position of exon 29, which was located at the upstream close neighbor nucleotide of classical donor splicing site (GT) of intron 29. Therefore, this position may play an important role as splicing modulator. The BDGP and the NetGene2 splice prediction programs were then employed to test whether this mutation could alter the splicing of the *TSC2* transcript. Both predictions resulted in the abolishment of the donor splice site. Finally, the in silico analysis by ASSP software showed that the splice donor site strength remarkably decreased from a score of 8.434 to 4.895 (cut off: 4.5).

**Fig. 7:**
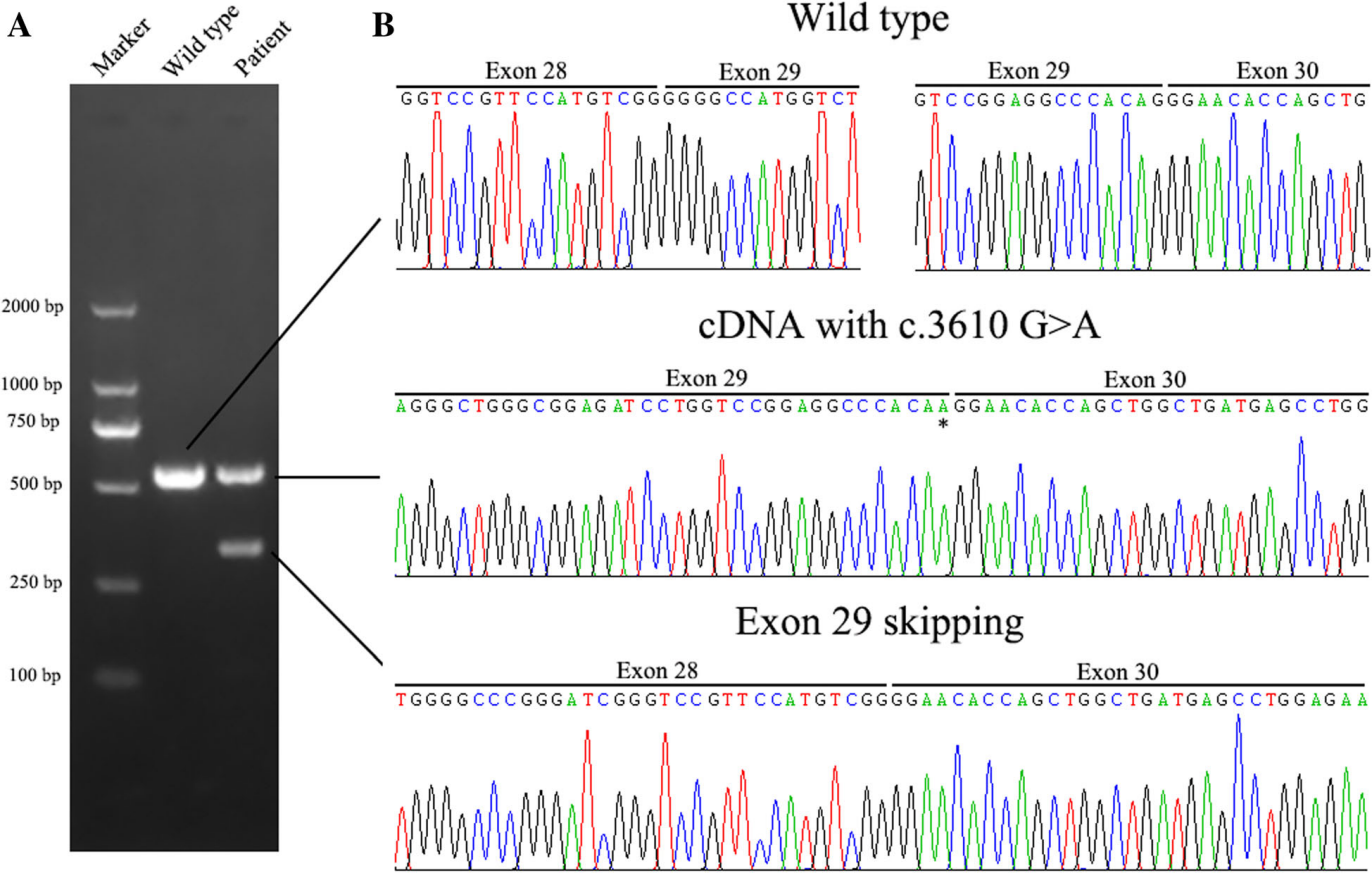
RNA analysis. **a** Electrophoresis for amplification of TSC2 cDNA in the proband and a normal person. **b** Sequencing chromatogram of TSC2 cDNA in the proband and a normal person. Upper panel, wild type; Middle panel, transcripts with missense mutation c.3610G > A; Lower panel, transcripts lacking exon 29. The asterisk indicated the position of mutation (c.3610G > A) of exon 29 in TSC2

The mini-gene assay performed to study the effects at the transcript level of the novel c.3610G > A variant showed that the wild-type gave rise to a 476 bp PCR product containing exon 29, the empty pSPL3 control gave rise to a 263 bp PCR fragment missing exon 29 of TSC2 gene. However, the c.3610G > A mutant construct produced two transcripts corresponding to 263 and 476 bp PCR fragments, respectively (Additional file 2: Figure S1). We then investigated whether the mutation c.3610G > A really led to exon 29 skipping in an actual patient. The cDNA was reverse transcribed from total RNA extracted from peripheral blood leucocytes of the proband. By cDNA sequencing, the exon 29-excluded transcript was identified only in the proband and not in a not-mutated individual. Approximately 44% of the cDNA products inserted into the pGEMT Easy vector were missing exon 29, whereas 56% were in-frame cDNA with the single nucleotide change c.3610G > A (Fig. 7).

## Discussion and conclusions

In this investigation, by preliminary mutation analysis of *TSC* gene in this family, a novel heterozygous mutation (c.3610G > A, p.Gly1204Arg) of exon 29 was identified in the *TSC2* gene. Predictions by in silico softwares revealed that it could be pathogenic in both protein and mRNA levels. On protein level, the substitution of a small molecular Gly to a more bulky Arg residue at highly conserved position 1204 strongly suggests that this missense mutation is pathogenic. On mRNA level, the results based on the mini-gene splicing assay and the RNA analysis from peripheral blood leucocytes confirmed the deleterious effect of the c.3610G > A alterations on the splicing process, demonstrating that the last nucleotide (c.3610G) of exon 29 in *TSC2* gene is an important controller of splicing. The change of guanine to adenine at this position led indeed to a significant decrease in the ability of donor splice site recognition. Above data strongly suggest that the exonic mutation of c.3610G > A disturbed the normal splicing. The skipping of whole exon 29 is predicted to lead to a defective tuberin with an in-frame deletion of 71 amino acids from p.Gly1133 to p.Thr1203 (p.Gly1133_Thr1203del), and a severe impairment of the normal hamartin-tuberin interaction, thus resulting in an upregulation of the mTOR pathway and the generation of TSC. Therefore, with combination of in silico algorithms, in vitro assays and in vivo analyses, an exonic point mutation, which may lead to the disease by both an exon skipping form (accounting for 44%) and a single amino residue alteration (accounting for 56%), was determined in the patients with TSC.

More than half of missense mutations in human are estimated to be the trigger for diseases via affecting the splicing process [13]. For years, only intronic mutations affecting donor or acceptor splice sites (DS or AS) were considered to potentially alter transcriptional processing. This has been questioned as the understanding of splicing mechanisms and concerned splicing signals has improved. To our knowledge, the core splicing signals were constitutive of the three motifs (DS, AS and branch point sequence BPS). However, it has been elucidated that the additional signals classified as exonic splicing enhancers (ESEs) or silencers (ESSs), which can promote or inhibit the exon recognition, respectively [14, 15]. In the same way, enhancer (ISE) or silencer (ISS) signals can also be located within introns. In view of the importance of core and auxiliary splicing signals in the pre-mRNA splicing process, it is now recognized that all types of mutations (missense, nonsense as well as small insertions or deletions) can indeed lead to splicing defects either by disrupting or creating signals [16].

In the present family, the proband was characterized by multi-systematic impairment and severe phenotypes, including severe AML of both kidneys, pulmonary LAM and retinal hamartoma. Her son (IIc), a highly suspicious TSC patient, died of epilepsy at the age of 6. Thus, consistent with previous study [7, 12, 17, 18], this family harboring the c.3610G > A *TSC2* mutation demonstrated a relatively severe phenotype. Furthermore, this investigation revealed that there was great phenotype variability and different penetrance among TSC patients who share the same mutation of *TSC2* gene. The “two-hit” hypothesis, loss of heterozygosity (LOH) or biallelic inactivation caused by a somatic “second-hit” mutation in the other unaffected TSC1 or TSC2 allele on the basis of a heterozygous germline mutation, is an important concept in understanding the variable clinical expression in TSC. However, it was not possible to confirm this hypothesis because we cannot obtain the pathological tissue of this patient. Additionally, modifier genes or other factors may be involved in the phenotype variability of TSC. More recently, some investigations revealed that epigenetic changes, including microRNAs that regulate TSC1 and TSC2 protein expression, may also contribute to phenotype diversity in TSC [19].

In this study, a novel *TSC2* missense mutation (c.3610G > A, p.Gly1204Arg), acting both at protein level as a missense alteration (56% of the transcript), and also at mRNA level contributing to exon 29 skipping (44% of the transcript), was identified in a Chinese family with typical TSC. This finding will further expand the TSC mutation database and increase the comprehension of the molecular abnormalities caused by TSC.

## Additional files

Additional file 1: Materials and methods. A detailed description of the sample acquisition, next generation sequencing (NGS), bioinformatics analyses, multiplex ligation-dependent probe amplification (MLPA), Sanger sequencing, mini-gene constructions expression and RNA analysis. (DOCX 28 kb)

Additional file 2: **Figure S1.** The mini-gene splicing assay based on the pSPL3 exon trapping vector. A. The pSPL3 vector contains the two exons, namely SD and SA, and a functional intron, with transcription beginning following the SV40 promoter and ending at the LPAS. Wild pSPL3-W and mutant pSPL3-M plasmids containing 293 bp of intron 28, 213 bp of exon 29 and 352 bp of intron 29 were separately cloned into the XhoI and NheI cloning sites of the pSPL3 vector. B. Agarose gel electrophoresis of RT-PCR products. SD6 and SA2 primers were designed for RT-PCR amplification of cDNA sequences generated by transfected 293 T cells. Lane 1: marker; Lane 2: 476 bp (263 + 213 bp); Lane 3: empty vector (263 bp); Lane 4: 263 and 476 bp (263 bp + 213 bp). MCS = Multiple cloning sites; LPAS = late poly(A) signal. (TIFF 2397 kb)

## Abbreviations

AML: Angiomyolipoma
AS: Acceptor splicing site
BPS: Branch point sequence
DS: Donor splicing site
ESEs: Exonic splicing enhancers
ESSs: Exonic splicing silencers
GTPase: Guanosine triphosphatase
ISE: Intronic splicing enhancer
ISS: Intronic splicing silencer
LAM: Lymphangioleiomyomatosis
mTORC1: mechanistic target of rapamycin complex
Rheb: Ras homolog enriched in brain
TSC: Tuberous sclerosis complex
