# Supplementary material for: Identification of a novel TSC2 c.3610G > A, p.G1204R mutation contribute to aberrant splicing in a patient with classical tuberous sclerosis complex: a case report
Source: BMC Med Genet. 2018 Sep 20;19:173. doi: 10.1186/s12881-018-0686-6 (PMC6149227; doi:10.1186/s12881-018-0686-6)
Supplement: Supplementary file 1 — Materials and methods. A detailed description of the sample acquisition, next generation sequencing (NGS), bioinformatics analyses, multiplex ligation-dependent probe amplification (MLPA), Sanger sequencing, mini-gene constructions expression and RNA analysis. (DOCX 28 kb) [file 12881_2018_686_MOESM1_ESM.docx]

**Identification of a novel TSC2 c.3610G>A, p.G1204R mutation contribute to aberrant splicing in a patient with classical tuberous sclerosis complex: a case report**

**Additional file 1:**

**Methods**

***Subjects***

The subjects were a Chinese family of five from Shandong province, China. The pedigree of the family is shown in Fig. 1. Blood samples were collected from all family members. And one hundred unrelated heathy subjects from the same ethnicity were selected to evaluate the mutation found in this study.

***Next*** ***Generation Sequencing (NGS)***

Genomic DNA was extracted from the peripheral blood of the patients and their family members by GenElute blood genomic DNA kit (Sigma, NA2010). A targeted NGS panel (Illumina HiSeq2000 platform, Inc., San Diego, CA, USA) was used to analyze the exon regions and flanking intronic regions of the 2 genes (*TSC1* and *TSC2*) associated with TSC. Generated reads were aligned to the human reference genome hg19 (University of California Santa Cruz (UCSC, http://genome.ucsc.edu) and National Center for Biotechnology Information b37.1 (NCBI). Reads that passed were then aligned to the human reference genome (UCSC hg19) using the Burrows-Wheeler Aligner (University of California, Santa Cruz, CA, USA). Single-nucleotide polymorphisms (SNPs) and small insertions/deletions were called with SAMtools (version 0.1.7). The variant call file (VCF) containing these variants was annotated with Variant Effect Predictor v83 and the dbNSFP (Database for Nonsynonymous SNPs’ Functional Predictions) v3.1. The Genome Analysis Toolkit was used to filter variants meeting the specified annotations (missense and predicted loss-of-function), inheritance (dominant, *de novo*, or parentally inherited modifier), and population allele frequency (based on the ExAC database, v0.3 release of 60,706 individuals).

***In silico analyses***

Three online programs (i.e. PolyPhen-2 <http://genetics.bwh.harvard.edu/pph2/>, SIFT http://sift.jcvi.org/, and Mutation Taster <http://www.mutationtaster.org/>) were employed to predict pathogenicity of the variants resulting from the NGS analysis. Moreover, for amino acid substitutions, multiple sequence alignments using tuberin orthologs of human (NM_000548.4), callorhinchus milii (XP_007908633), macaca mulatta (XP_014980835), chimpanzee (XP_016784109), house mouse （NP_001273642）, rat (NP_036812), xenopus tropicalis (NP_001123795), zebrafish (NP_001315330) by using Vector NTI Advance 10-Align were used to evaluate evolutionary conservation.

The BDGP (available at <http://www.fruitfly.org/seq_tools/splice.html>), the NetGene2 (available at <http://www.cbs.dtu.dk/services/NetGene2/>) and ASSP (available at <http://wangcomputing.com/assp/>) splice prediction programs were finally employed to test the effect of DNA variants on the splicing process.

***Multiplex ligation-dependent probe amplification (MLPA)***

MLPA was performed to verify copy number changes in *TSC1* and *TSC2* genes by the SALSA MLPA kit P046 vs. 04, covering 31/41 *TSC2* exons, and by the SALSA MLPA kit P124 vs. 01, covering 16/23 *TSC1* exons. The experiment was performed following the manufacture’s instructions.

***Sanger sequencing***

The potential candidate variant identified by NGS in the *TSC2* gene was validated by Sanger sequencing. The TSC2 reference sequence (NG_005895) was obtained from the Genbank. The target site of c.3610 G>A and the flanking sequences from each family member were amplified with specific primers (Forward: 5'-GCCTGCTCTGGGTGCTGGTGTTTCC-3'; Reverse: 5’-CCGAGATTGCGCCACTGCGTTCC-3’; product=683 bp). PCR products were sequenced using an ABI Prism 3700 DNA Analyzer (Applied Biosystems, Calif., USA). Sequence analysis was finished through in *silico* software Chromas 2.31 and Vector NTI Advance 11.5.

***Mini-gene Constructions and Expression***

To investigate the effect on the splicing process of the c.3610G>A variant, *in vitro* analysis was performed using a mini-gene splicing assay based on the pSPL3 exon trapping vector [1,2]. The fragments with the wild or mutant alleles involving exon 29 (213bp), flanked by upstream intronic sequence (293bp) and downstream intronic sequence (352bp), were cloned into the splicing vector pSPL3 using specific primers (forward, 5’-GCCT­­GCTCTGGGTGCTGGTGTTTCC-3’; reverse, 5’-GGCGGATCACGAGGTCAGGAGATTGA-3’) linking the XhoI and NheI restriction enzyme sites (TGGAGC^TCGAG: XhoI; AATTTG^CTAGC: NheI) (Additional file 2: Fig. 1). The ancestral and mutant type constructs were named pSPL3-W and pSPL3-M, respectively. All constructs were verified to contain the correct sequence by direct sequencing. Human epithelial kidney 293 T (HEK 293 T) cells were cultured in DMEM medium containing 10% fetal bovine serum (FBS), penicillin (100 U/L), and streptomycin (100 mg/L) at 37 °C in a 5% CO_2_ atmosphere. One day before transfection, cells were transferred to 6-well culture plate to grow to approximately 70% to 80% confluence in an antibiotic free medium. Cells were then transfected with 4 μg plasmid DNA (pSPL3-W, pSPL3-M and empty pSPL3-control each) using OPTI-MEM® IMedium and Lipofectamine 2000 (Invitrogen, Carlsbad, CA, USA) according to the manufacturer’s instructions. Cells were harvested, total RNA was extracted after 24 h transfection with the RNAsimple Total RNA Kit (Tiangen, Beijing, China) and used for RT-PCR to confirm the splicing patterns. First-strand cDNA was synthesized from 2 to 3 μg of total RNA by random-primed reverse transcription with Superscript II Reverse Transcriptase (Invitrogen Corporation, Carlsbad, CA). To evaluate the pattern of transcripts from the transfected minigenes, the following vector-specific primers were used for RT-PCR amplification: a forward primer SD6 (5’-TCTGAGTCACCTGGACAACC-3’) and a reverse primer SA2 (5’-ATCTCAGTGGTATTTGTGAGC-3’).

The PCR amplification reaction was performed as follows: in 50 μl volume, 2 μl of cDNA, 5 μl of Expand High Fidelity buffer 3 (Roche, Mannheim, Germany), 1 μM of each primer, 0.8 μlM dNTPs, and 2.6 U Expand High Fidelity enzyme mix (Roche, Mannheim, Germany) in a 9700 (Applied Biosystem, Foster City, CA, USA) thermal cycler. Thermal conditions were 30 cycles of 95 °C for 30 seconds, 58 °C for 30 seconds, and 68 °C for 1 minute, preceded by 2 minutes at 95 °C, and followed by a final elongation step at 68 °C for 10 minutes. The PCR products were separated by electrophoresis on a 3% agarose gel. All transcripts were analyzed by sequencing.

***RNA analysis***

cDNA was reverse transcribed from total RNA extracted from peripheral blood leucocytes. Splice mutations were detected by cDNA-sequencing using one pair PCR primers spanning exon 28 to exon 30. The primers used for the PCR analysis were 5′-CCCGGAGTCGAGCTCCAGC -3′ and 5′-GTGTTGGAGCGAGGCAGAGGA -3′. To identify the relative amounts of different transcripts, the cDNAs were amplified with 22 cycles and then ligated into the pGEM-T Easy vector and 48 of the resulting colonies were analyzed by direct sequencing. In addition, amplification products with 33 cycles were separated on 2.5% TAE/agarose gels.

**References**

1. Wang X, Zhao X, Wang X, Yao J, Zhang F, Lang Y, et al. Two Novel HOGA1 Splicing Mutations Identified in a Chinese Patient with Primary Hyperoxaluria Type 3. Am J Nephrol. 2015; 42:78-84.

2*.* Bottillo I, De Luca A, Schirinzi A, Guida V, Torrente I, Calvieri S, et al. Functional analysis of splicing mutations in exon 7 of NF1 gene. BMC Med Genet. 2007; 8:4.
