# Supplementary figures and images for: Identification of a novel TSC2 c.3610G > A, p.G1204R mutation contribute to aberrant splicing in a patient with classical tuberous sclerosis complex: a case report
Source: BMC Med Genet. 2018 Sep 20;19:173. doi: 10.1186/s12881-018-0686-6 (PMC6149227; doi:10.1186/s12881-018-0686-6)

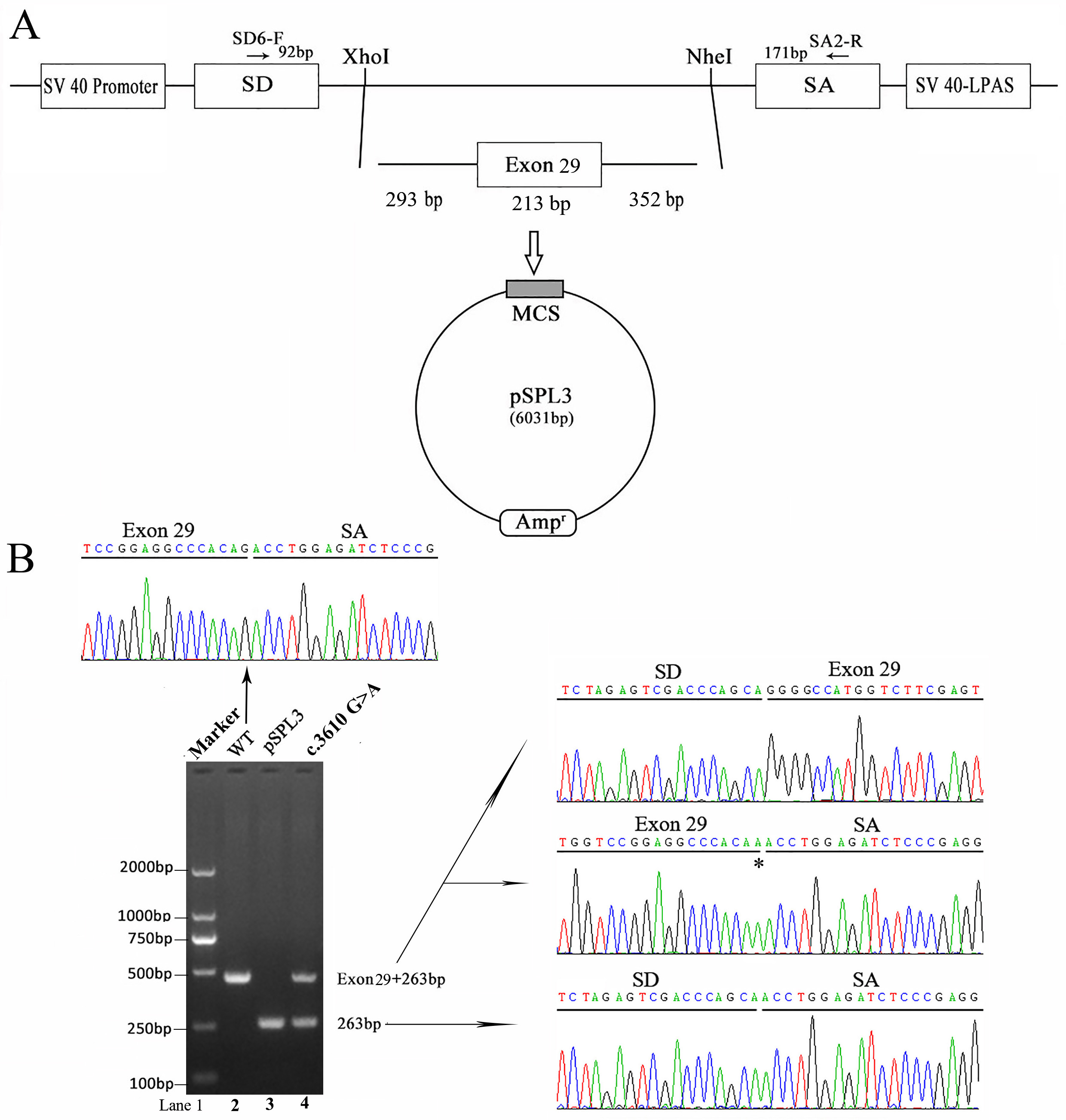

Supplement: Supplementary file 2 — Figure S1. The mini-gene splicing assay based on the pSPL3 exon trapping vector. A. The pSPL3 vector contains the two exons, namely SD and SA, and a functional intron, with transcription beginning following the SV40 promoter and ending at the LPAS. Wild pSPL3-W and mutant pSPL3-M plasmids containing 293 bp of intron 28, 213 bp of exon 29 and 352 bp of intron 29 were separately cloned into the XhoI and NheI cloning sites of the pSPL3 vector. B. Agarose gel electrophoresis of RT-PCR products. SD6 and SA2 primers were designed for RT-PCR amplification of cDNA sequences generated by transfected 293 T cells. Lane 1: marker; Lane 2: 476 bp (263 + 213 bp); Lane 3: empty vector (263 bp); Lane 4: 263 and 476 bp (263 bp + 213 bp). MCS = Multiple cloning sites; LPAS = late poly(A) signal. (TIFF 2397 kb) [file 12881_2018_686_MOESM2_ESM.tiff]
